# Molecular and immune heterogeneity in synchronous melanoma metastases

**DOI:** 10.1186/2051-1426-3-S2-P262

**Published:** 2015-11-04

**Authors:** Alexandre Reuben, Christine Spencer, Jason Roszik, John Miller, Lawrence Kwong, Hong Jiang, Cara Haymaker, Pei-Ling Chen, Jacob Austin-Breneman, Whijae Roh, Latasha Little, Yu Cao, Haven Garber, Marie-Andrée Forget, Vancheswaran Gopalakrishnan, Rodabe Amaria, Michael Davies, Chantale Bernatchez, Edwin Roger, Parra Cuentas, Jaime Rodriguez, Michael Tetzlaff, Scott Woodman, Karen Dwyer, Padmanee Sharma, James Allison, Lynda Chin, Andrew Futreal, Zachary Cooper, Jennifer Wargo

**Affiliations:** 1 The University of Texas MD Anderson Cancer Center, Houston, TX, USA

## 

Despite recent advances in the treatment of metastatic melanoma through targeted and immunotherapy, the majority of patients do not achieve a durable response. Research efforts to better understand responses are underway, and numerous molecular mechanisms of resistance to targeted therapy have been identified. There is a growing appreciation of genomic heterogeneity as a contributor to resistance to therapy, although immune heterogeneity has not been well characterized. The goal of the present study is to better understand genomic and immune heterogeneity in synchronous metastases within melanoma patients, with the potential to identify actionable strategies to overcome resistance. In this study, we prospectively evaluated 36 tumors from 16 melanoma patients (n=5 treatment-naïve, n=6 targeted therapy, n=5 immunotherapy). Distinct synchronous metastases were evaluated by whole exome sequencing and NanoString analysis and showed up to 36% tumor-specific mutations as well as significant differences in expression of immune pathway effectors. Accordingly, we performed immune profiling by flow cytometry and immunohistochemistry demonstrating significant immune heterogeneity between synchronous melanoma tumors in all patients, most notably in the CD4^+^ and CD8^+^ T cell compartment. Deep TCR sequencing data revealed that T cell populations infiltrating synchronous metastases presented different specificities, with less than 10% of T cell clones shared between 2 tumors in the same patient. Additionally, the NetMHC 3.4 algorithm revealed that 10-30% of predicted neoantigens were unique to individual tumors and that over 10% of these presented high HLA-binding affinity. Together, these data suggest significant genomic and immune heterogeneity between synchronous metastases in melanoma patients – not only in the setting of therapy but also prior to its initiation. This has important clinical implications, and could help explain variable responses to therapy, however this hypothesis must be tested carefully in a larger data set. Nonetheless, these findings may have significant implications for the treatment of melanoma and other cancers.

